# Incorporating Intracellular Processes in Virus Dynamics Models

**DOI:** 10.3390/microorganisms12050900

**Published:** 2024-04-30

**Authors:** Stanca M. Ciupe, Jessica M. Conway

**Affiliations:** 1 Department of Mathematics, Virginia Polytechnic Institute and State University, Blacksburg, VA 24060, USA; 2 Department of Mathematics and Center for Infectious Disease Dynamics, Penn State University, State College, PA 16802, USA

**Keywords:** mathematical modeling, viral dynamics model, intracellular dynamics, viral life cycle

## Abstract

In-host models have been essential for understanding the dynamics of virus infection inside an infected individual. When used together with biological data, they provide insight into viral life cycle, intracellular and cellular virus–host interactions, and the role, efficacy, and mode of action of therapeutics. In this review, we present the standard model of virus dynamics and highlight situations where added model complexity accounting for intracellular processes is needed. We present several examples from acute and chronic viral infections where such inclusion in explicit and implicit manner has led to improvement in parameter estimates, unification of conclusions, guidance for targeted therapeutics, and crossover among model systems. We also discuss trade-offs between model realism and predictive power and highlight the need of increased data collection at finer scale of resolution to better validate complex models.

## 1. Introduction

The first mathematical models to gain insight into in-host viral infection were developed in the late 1980s/early 1990s, following on the heels of the discovery of the human immunodeficiency virus (HIV) [1,2,3,4]. The so-called “standard model”, derived from these early efforts and based on predator–prey dynamics, saw success in the mid-1990s when its validation against HIV viral load data from patients undergoing antiretroviral therapy allowed for the quantification of in-host dynamical quantities, such as the infected cell death rate [5]. Since that time, the standard model and its extensions have been used to further describe HIV [6,7,8,9,10,11,12,13,14,15,16,17] and a series of other viral infections, with pathogens such as hepatitis C (HCV) [18,19,20,21], hepatitis B (HBV) [22,23,24,25,26,27,28], influenza [29,30], ebola [31], and SARS-CoV-2 [32,33,34,35,36,37,38].

The standard model, which we introduce properly below, is relatively low-dimensional. While this was appropriate given the scarcity of early in-host viral infection data, with the initial models relying on only plasma viral load measurements for validation [5], some scientific questions are inaccessible via this simplified approach. Integrating intracellular processes within cell population-level viral dynamic models can yield more biologically realistic representations of the virus–host interactions. Puzzles, such as uncovering mechanisms explaining rapid hepatitis C virus loss under direct acting antivirals (DAAs), are only accessible if we consider intracellular interactions [39]. In this review, we focus on multiscale models of viral dynamics, where the scales in consideration represent virus–cell interactions and intracellular dynamics. At the intracellular scale, we emphasize modeling of the viral life cycle. For reviews of a systems biology/scale of molecular interactions, the reader is referred to [40,41].

We focus this review on two principle contributions to the advancement of knowledge made through the addition of intracellular dynamics within virus dynamics models. First, we summarize studies that show improved insights into viral pathogenesis and infection dynamics under intracellular considerations. For a simple example, consider the in-host basic reproduction number $R_{0}$, which is the number of infected cells (or virus particles) that are produced by one infected cell (or virus particle), when the virus is introduced into a population of uninfected target cells [42,43]. It has been shown that incorrectly accounting for the period of cellular infection before an viral production may yield wildly inaccurate $R_{0}$ values [44], which in turn can yield poor predictions of treatment efficacy in reducing $R_{0}$. Second, we summarize studies highlighting the role of intracellular considerations in streamlining treatment strategies and guiding drug development. For an example, consider the case of DAAs therapy which successfully controlled hepatitis C virus, leading to cure. It was shown that ignoring intracellular interactions yielded an overestimate of HCV viral life-span and resulted in incorrect hypotheses for different drug efficacy and drugs mode of action (MOA) [39,45]. As modern antiviral drugs that target specific stages of the life cycle of different viruses are being developed, models considering intracellular events are also needed. They are essential tools for investigating the impact of antiviral therapies on viral replication and clearance [39,46]. Furthermore, they can provide guidance on potential treatment approaches, such as timing, frequency, and duration. Ideally, these models can inform the early stages of drug development by identifying targets, hypothesizing different mechanisms of action, quantifying efficacy of monotherapy, and suggesting instances where there is need for combination therapy.

We structure this review paper as follows. In the first part, we provide an overview of intracellular modeling approaches as they arise naturally from the standard viral dynamics model, noting mathematical and methodological advances, and associated results. While the models, methods, and techniques are intended to be generic, they are presented in the context of the different viral infections that inspired their development. In the second part, we focus on several key examples where inclusion of intracellular modeling in studying hepatitis B, hepatitis C, hepatitis D, influenza, and HIV infections led to scientific discoveries. These stories are not meant to be exhaustive but, rather, serve as bellweather examples of the power and impact of intracellular modeling. We conclude with a discussion on the trade-off between model complexity and data availability. This could serve as a call for strengthening interactions between modelers and experimentalists, with the aim of more effectively collecting the type of data needed for model validation and to further ensure scientific understanding and progress.

## 2. Modeling Viral Dynamics: The Standard Model

The standard model of viral dynamics tracks the spread of the virus between cells within an infected individual. The model describes the interactions between target cells *T*, infected cells *I*, and virus *V* as follows. Target cells *T* enter the system at rate λ, die at rate *d*, and are infected by virus *V* with mass-action infectivity rate β. Infected cells *I* die at rate δ (δ>d) and produce virus at rate *p*. Virus is cleared at rate *c*.

These interactions are described by the following ODE system:

(1) $$\begin{array}{cc} \frac{dT}{dt} & =\lambda -dT-\beta TV,\\ \frac{dI}{dt} & =\beta TV-\delta I,\\ \frac{dV}{dt} & =pI-cV. \end{array}$$

Model Equation (1) with source and death terms (λ>0 and d>0) holds the implicit assumption that the timescale of the viral infection is long, predicting that an established infection results in persistent (chronic) disease. Short-lived (acute) viral infections, where an established infection is eventually cleared, have been modeled by setting λ=d=0; examples include influenza [29,47], dengue [48,49,50,51], Zika [52], oncolytic adenoviruses [53], and SARS-CoV-2 [32,33,34,35,36]. For reviews of in-host mathematical models of acute and chronic viral infection, see [30,42,54,55].

Model Equation (1) was used to gain insight into HIV viral dynamics following the initiation of antiretroviral therapy (ART). At the beginning of the HIV epidemic, ART consisted of combination therapy with protease inhibitors (PIs) and reverse transcriptase inhibitors (RTs) drug classes [5,56]. Under ART, patients’ HIV RNA showed delayed response followed by biphasic decay. To address the observed dynamics, model Equation (1) was modified to account for interference with virus production by replacing *p* with (1−ϵ)p and/or for reduction in new infections by replacing β with (1−η)β, where 0≤ϵ≤1 and 0≤η≤1 are drug efficacy. The mathematical model becomes:

(2) $$\begin{array}{cc} \frac{dT}{dt} & =\lambda -dT-(1-\eta )\beta TV,\\ \frac{dI}{dt} & =(1-\eta )\beta TV-\delta I,\\ \frac{dV}{dt} & =(1-\epsilon )pI-cV. \end{array}$$

**Figure 1:**
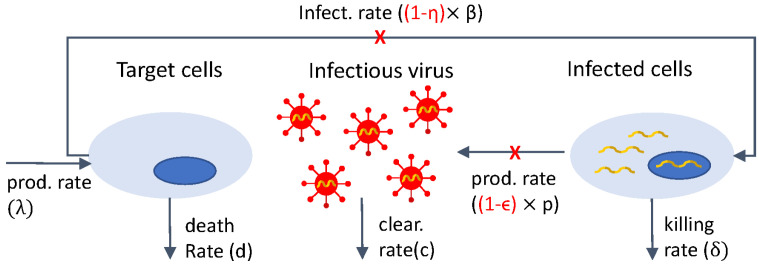
Diagram for model Equation (2).

A schematic representation of model Equation (2) is shown in Figure 1. The same schematic with ϵ=η=0 represents the interactions in model Equation (1).

In a first study, model Equation (2) with η=0 and ϵ=1 was fitted to plasma HIV RNA titers under PIs (ritonavir) therapy alone, resulting in an initial estimate for the virus clearance rate, *c* [5].

One drawback of the early modeling studies was the limited amount and type of data used for validation, which mainly consisted in plasma viral titers [5,18,56,57]. As the ability to collect empirical data within a cell increased, the field of virus dynamics evolved as well, allowing for intracellular events to be considered, and consequently incorporated into models Equations (1) and (2). The added complexity allowed for detailed mechanistic insight into the infection process, such as viral entry, replication, transcription, translation, and assembly within host cells. It addressed the impact of cellular coinfection. It enabled the study of host immune responses at the cellular level. Lastly, it allowed for investigation of the impact of antiviral drugs at various stages of the viral life cycle. Depending on the nature of the scientific question, the paucity of data, computational resources, and the level of detail required, these models include incremental or extensive details. In the next sections, we will provide an overview of existing models of intracellular viral dynamics, motivating the choice for simplicity or complexity.

## 3. Modeling Viral Eclipse Phase

The basic models Equations (1) and (2) assume instantaneous production of new virus by infected cells. However, intracellular events, such as translation of the viral genome, induce delays between the time of cell infection and the time of viral production. A standard adjustment to models Equations (1) and (2) is the assumption of an “eclipse phase”, which refers to the stage in the viral life cycle where the cell is infected but newly produced viruses are not yet being released from the infected host cell. Neglect of these intracellular delays can result in underestimates in the basic reproduction number $R_{0}$ [44,58] and, consequently, biased estimates of treatment efficacy.

Inclusion of the eclipse phase has taken two forms. The first one considers different distributions to model the delay in viral production, and the second one expands models Equations (1) and (2) by adding one (or many) explicit compartments for the eclipse stage(s). The two approaches are closely related, with the second approach with infinitely many stages and fixed mean total duration across all stages converging into the first approach for a delta-distributed, i.e., fixed, delay. Here, we will briefly explain each approach. We will limit ourselves to incorporating the eclipse phase into model Equation (1), with changes to model Equation (2) following a similar approach.

When a delay τ is incorporated between cell infection and viral production, model Equation (1) becomes:

(3) $$\begin{array}{cc} \frac{dT}{dt} & =\lambda -dT-\beta TV,\\ \frac{dI}{dt} & =\beta \int_{0}^{t}f\left(\tau \right)T\left(t-\tau \right)V\left(t-\tau \right)e^{-m\tau }\;d\tau -\delta I,\\ \frac{dV}{dt} & =pI-cV. \end{array}$$

Here, f(τ) is a distribution describing intracellular delays between cell infection and the formation of productively infected cells, and $e^{-m\tau }$ accounts for the loss of an infected cell between initial infection and viral production. This latter term is often neglected, as we expect *m* to be small, to account for longer timescale of natural cell death compared to pathogen-induced cell death.

The fixed delta-distributed delay, f(t)=δ(t−τ) for delay τ, has been used in models of influenza and HIV infections [59,60,61,62,63]. Herz et al. used model Equation (3) (with the added therapy rates η and ϵ) to estimate parameters specific to HIV and HBV infections [60]. They showed that, given frequent plasma HBV DNA titers, one can estimate the free virus clearance rate (parameter *c*) and, consequently, the corresponding HBV half-life. However, given the rapid clearance of HIV (on the same timescale of pharmacological delays—defined as the time for drug to take full effect), one can only reasonably estimate the turnover rate of productively HIV-infected cells. This led to the conclusion that the first shoulder of plasma HIV RNA titers accounts for a complicated combination of pharamcological delays, intracellular delays, decay of infected cells, and viral clearance [60]. This was in contrast with model Equation (2)’s prediction which attributed the initial HIV RNA shoulder to pharmacological and intracellular delays alone [5].

We focus primarily on models with a common assumption that cell infectivity is described by mass action, that is, that the rate of infection is proportional to the target cell and the virus populations at rate βTV. This is the preferred incidence rate and is well tested by data. Alternative cell infectivity rates have been considered, expressing infection saturation at high viral loads, including Holling type 2 response βTV/(1+bV) [64], and variations such as the Beddington-Angelis infection rate βTV/(1+aT+bV) [65] and the Crowley–Martin infection rate, βTV/(1+aT)(1+bV) [66,67,68]. These modeling approaches have been employed in viral dynamics models, including models accounting for delays describing intracellular dynamics, in addition to immune and latent cell dynamics. Though to our knowledge, models using these infectivity rates have not been validated with data, their theoretical properties, particularly in the case of fixed delays, have been well explored [69,70,71,72,73].

Fixed delays (as described by model Equation (3)) are technically challenging to implement and tend to induce oscillations when they are large enough, which is not observed in the data. Nonetheless, important insights have been derived from such models. Ribeiro et al. used a fixed-delay model to estimate the basic reproduction number $R_{0}$ (≈eight for in-host HIV infection) [58]. More recently, Lord et al. used fixed delays to investigate HIV evolution, demonstrating that the length of the delay before apoptosis is an important trait, with apoptotic strategies being favored if apoptosis is rapid and viral budding strategies being favored if apoptosis is slow [59]. Lastly, Dixit & Perelson modified Equation (3) to directly incorporate pharmacokinetics into HIV models, thereby decoupling pharmacodynamic and intracellular delays [74,75]. Using plasma HIV RNA data from participants in a ritonavir study, they estimated an intracellular delay of approximately one day, in agreement with in vitro experiments and previous ad hoc calculations [74]. Using this estimate of intracellular delay, the authors showed that the average HIV generation time is ∼two days, resulting in ∼180 replication cycles per year. This result suggested that the replication rate of HIV is quite large, explaining the rapid emergence of drug-resistant HIV variants during mono- or two-drug therapy [74,76,77].

Mittler et al. [78] used a variation of model Equation (3) and compared it to empirical HIV RNA data following initiation of protease inhibitors. They investigated a variety of intracellular delay distributions f(t) and demonstrated that model-derived parameter estimates are not sensitive to the underlying delay distribution [78]. More recently, however, Kakizoe et al. found that an Erlang distribution resulted in improved fit when model Equation (3) is compared to in vitro SHIV-KS661 viral data. The estimate of intracellular delay, however, is one day [79], identical to intracellular delay estimates for other types of distributions used to explain plasma HIV RNA data [74].

When the choice for delay distributions f(t) is a gamma distribution, Equation (3) can be converted from an integro-differential system into a set of ordinary differential equations, with the eclipse phase being included as additional cellular compartment(s). For a single eclipse phase, the model becomes:

(4) $$\begin{array}{cc} \frac{dT}{dt} & =\lambda -dT-\beta TV,\\ \frac{dE}{dt} & =\beta TV-\gamma E,\\ \frac{dI}{dt} & =\gamma E-\delta I,\\ \frac{dV}{dt} & =pI-cV, \end{array}$$

where *E* represents a class of exposed cells, corresponding to infected cells who are not yet infectious, and 1/γ is the time spent in the exposed class. This is the second approach to modeling intracellular delays. The advantage of model Equation (4) is ease of implementation. Since parameter estimates are not sensitive to the underlying delay distribution [78], Equation (4) is the preferred model to account for single eclipse phase in respiratory synctial virus [80] and influenza infections [81]; and for multiple eclipse phases in generic viral infection [44] and ebola virus [31].

## 4. Modeling Cellular Coinfection

Cellular infection with multiple virions can yield distinct biological outcomes from infection with a single virion [82]. An important set example derives from viral infections of the liver, i.e., hepatitis viruses, where liver cells (hepatocytes) are the target cells. The presence of two or more replicating organisms in the same host can occur when HBV and HCV [83] or HBV and hepatitis D virus (HDV) (a satellite virus of HBV whose intracellular products are required for the completion of the HDV life cycle) [84] are present in the same individual. Infection with two pathogens is referred to as coinfection when it happens simultaneously, and superinfection when an individual with pre-existing hepatitis infection is infected with a different hepatitis virus. HBV-HDV coinfection leads to acute self-limiting hepatitis, with most individuals recovering fully [85]. By contrast, HBV-HDV superinfection leads to chronic disease and increased risk of disease progression, including cirrhosis and hepatocellular carcinoma [85]. Very few mathematical modeling studies of hepatitis B and C coinfection have been developed [86]. Conversely, there are several mathematical modeling studies investigating HBV-HDV coinfection and superinfection [87,88,89,90]. They are extensions of the standard model Equation (1) through addition of compartments representing concentration of cells infected with two viruses. Using these models, Goyal et al. showed that, compared to HBV mono-infection, dual HBV-HDV infection resulted in lower chronic HBV DNA levels, with more marked HBV DNA suppression for HBV-HDV coinfection compared to HBV-HDV superinfection [87]. Packer et al. showed that infected cell proliferation may play a significant role in chronic HDV infection [90]. Lastly, since HDV requires hepatitis B surface antigen (HBsAg) for its assembly and release, most HDV therapeutics have focused on the role of the drugs in inhibiting HBsAg production and secretion. As a result, mathematical models of HDV therapy have focused on understanding drugs mode of action [91,92,93]. In an early study, Guejd et al. fitted mathematical models to HBsAg and HDV RNA data from HBV-HDV coinfected patients receiving pegylated-interferon (peg-IFN) therapy. They found that the main effect of peg-IFN is to reduce HDV RNA production and release and that the HBsAg-productive-infected cells are the main source of HDV RNA production [91]. In another study, Shektman et al. used a mathematical model to show that the rapid declines in both HBsAg and HDV RNA following monotherapy with the nucleic acid polymer (REP 2139-Ca) is due to the inhibition of HBsAg production and of HDV RNA replication [92]. Recently, Shektman et al. investigated the dynamics of HDV RNA under therapy with an entry-inhibitor (bulevirtide (BLV)) monotherapy in three patients. They discovered that, while the BLV assumed mode of action in blocking the HDV RNA entry/infection explains data in two patients, it does not explain the data in the third. In this patient, a transient increase in HDV RNA was seen during the first four weeks of treatment. To explain this transient increase (seen also in BLV monotherapy in HBV monoinfection), they hypothesised that blocking the HDV DNA entry into the liver has a secondary effect of reducing HBV DNA clearance by the liver [89].

Cellular coinfection is also a frequent occurrence outside of viral infections of the liver, with viruses inducing chronic infection such as HIV [94,95] and acute infections such as influenza [96,97,98]. It has been predicted that as high as ten percent HIV-infected cells are multiply infected [99,100]. The standard model Equation (1) and its variations employ mass-action infectivity rates to account for cell mono-infection. Explicit modeling of cellular coinfection events is critical for understanding how multiplicity of infection (MOI) impacts estimates for infected cell death and viral production rates [101], the evolution of drug-resistant variants [102], and immune escape [103,104].

With few exceptions (e.g., [105]), models used to investigate coinfections are extensions of the standard model Equation (1) through addition of compartments representing concentration of doubly- and triply-infected cells, etc. [74,75,104,106,107]. While theoretical results are accessible in these models, their high-dimensional nature renders them challenging to use when they are validated against plasma virus titer data, due to the large number of unknown parameters.

The standard model Equation (1) and its descendants assume that the host cell resources are limiting, and, thus, viral production rate and burst size are independent of the extent of cellular coinfection [75]. Since this assumption may not hold across all viral pathogens, Koelle et al. [108] considered a different approach for modeling cellular coinfection. They developed an alternate class of low-dimensional in-host model whose structure allows for cellular coinfection, based on the structure of epidemiological macroparasite models [109]. In its most general form, the model is described by the following ODE system:

(5) $$\begin{array}{cc} \frac{dH}{dt} & =\lambda -dH-H\sum_{i=0}^{\infty }\alpha_{i}p_{i},\\ \frac{dV}{dt} & =H\sum_{i=0}^{\infty }\gamma_{i}p_{i}-cV-\beta HV,\\ \frac{dP}{dt} & =\beta HV-dH\sum_{i=0}^{\infty }ip_{i}-H\sum_{i=0}^{\infty }i\alpha_{i}p_{i}, \end{array}$$

where *H* represents all target cells, both infected and uninfected, as both are targets for further infection; *V* is free (extracellular) virus; and *P* is the total amount of internalized virus across all target cells *H*. Note that *P* does not have an analog in the standard model. $p_{i}$ is the probability that a target cell is infected with *i* virions. As with the standard model, target cells are produced at constant rate λ and have per capita background mortality rate *d*. Infection-induced target cell death is modeled separately, with $\alpha_{i}$ the death rate of target cells infected with *i* virions. Note that background mortality and infection-induced cell death both result in the loss of internalized virus, *P*. New virions are produced at rate $\gamma_{i}$ by cells infected with *i* virions, are cleared at rate *c*, and infect new cells with mass-action infectivity β; note that here, the interpretation of βHV is the free virion loss and also increase of internalized virus due to cell entry of free virus. If one assumes that cellular MOI has a negative binomial distribution (as in the standard model Equation (1)), the series can be summed.

Fitting model Equation (5) (together with terms for innate immune responses and the neglect of target cell limitation assumption) to influenza viral load resulted in statistically significant improvements compared to the standard model Equation (1) (with λ=d=0) [108]. Subsequent studies have used this novel paradigm for cellular coinfection to predict the shape of the interferon response to influenza infection [101,110,111] and to account for cellular coinfection in the context of acute HIV studies [112].

## 5. Modeling Viral Life Cycle: A Few Stories

**Figure 2:**
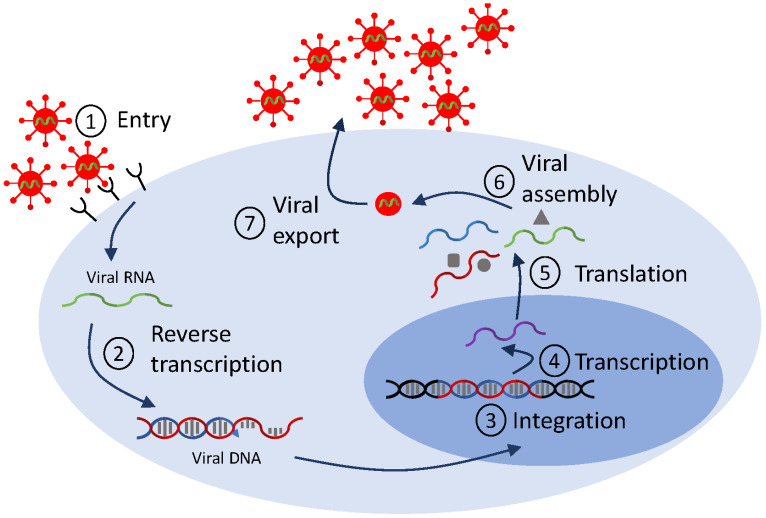
Diagram for a generic life cycle for RNA viruses. The checkpoints reference stages that are used in mathematical models.

**Figure 3:**
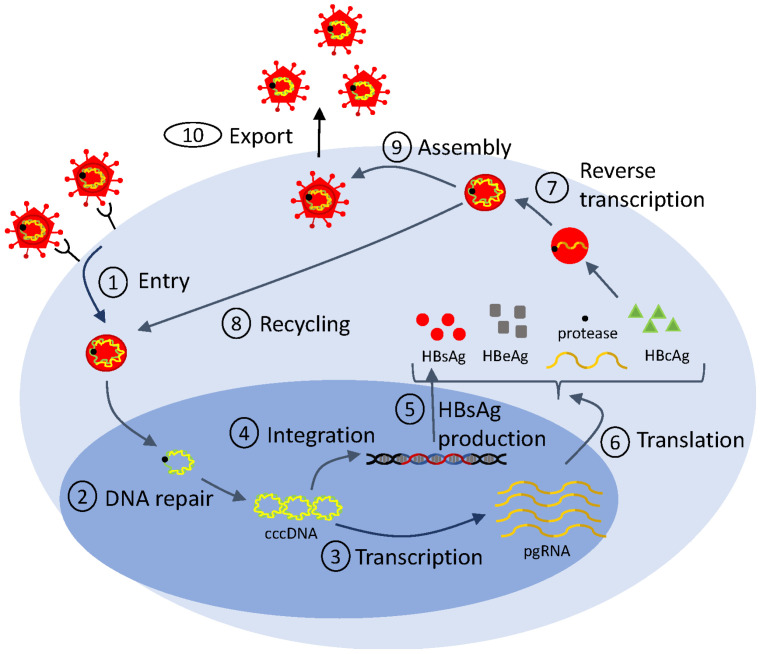
Simplified life cycle diagram used to model HBV dynamics and therapy.

Mathematical models of viral life cycle are useful tools for understanding the molecular mechanisms of viral replication, transcription, translation, integration, and assembly within the infected cell. They can be used to gain insights into intracellular processes. They can help identify antiviral targets [113,114], predict antiviral mode of action, and optimize interventions. They include several layers of detail, based on the biological question at hand and on the availability of data for validation. In Figure 2, we show a generic representation of the viral life cycle for RNA viruses (which, in this review, will be restricted to HIV, hepatitis C virus and influenza virus) and in Figure 3, we show a representation of the viral life cycle for one DNA virus, the hepatitis B virus. The two schematics will be used to explain choices for details in complex mathematical models.

In the next sections, we will present a few approaches taken for modeling intracellular interactions based on viral life cycle. We will also discuss approaches taken for modeling drug therapy, both within the standard model Equation (1) and within the intracellular models. Our examples will be restricted to three viral infections: HIV, hepatitis C virus, and hepatitis B virus.

### 5.1. Intracellular Modeling of HIV Infection and Therapy

Emergence of additional scientific questions, novel therapeutics, and more complex data dynamics required extensions of the standard therapy model Equation (2), mainly to incorporate more detailed biological interactions. An important set of examples were motivated by observations of HIV RNA dynamics under raltegravir, the first integrase inhibitor (FDA approval 2007) [115], a class of ART drugs that prevent HIV DNA integration into the host cell genome (checkpoint ➂ in Figure 2). Upon initiation of ART, viral loads decline; puzzlingly, raltegravir produces a steeper initial decline than other ART drugs. To better understand determinants of first-phase HIV decay, Gilmore et al. [116] explicitly modeled stages of the HIV viral life cycle in CD4+ T cells, including viral entry, reverse transcription, integration, and viral production (checkpoints ➀–➆ in Figure 2) as compartments, further extending the eclipse phase model Equation (4). This extended model showed that the observed differences across drug classes in the initial HIV RNA decline arise from increased death of infected cells when drugs enter productively infected cells post-integration, while, prior to integration, the half-lives of infected cells are similar to those of uninfected cells [116]. Simplifying the picture partway, Cardozo et al. [46] included pre- and post-integration compartments, and used them to quantify the efficacy of raltegravir, a great first step in determining the potency of integrase inhibitors in HIV infections.

These models have been expanded further and used for other scientific investigations. In particular, together with macaques data, they hypothesized that CD8+ T cells have both a cytolytic effect on infected cells before viral integration, and a direct, noncytolytic effect by suppressing viral production [117]. This result was an important contribution in our understanding of the role of CD8+ T cells [118,119].

Modeling investigations of APOBEC3G provide another set of studies solidifying the importance of intracellular detail into models of HIV infection to gain insight into therapeutics. APOBEC3G is an intracellular enzyme known to be an inhibitor of HIV infection. Its primary mode of inhibition is hypermutation of the viral genome (checkpoint ➁ in Figure 2), with mutational frequency of up to 10%, in a process that first requires APOBEC3G packaging into viral particles (checkpoint ➅). The HIV viral protein Vif protects the virus, essentially by binding to APOBEC3G and causing its degradation. In a pair of studies, Hosseini and Mac Gabhann investigated the antiviral potential of APOBEC3G and identified optimum therapeutic approaches via multiscale, intracellular models. They predicted that stem cell therapy resulting in a high fraction of APOBEC3G-overexpressing CD4+ T cells can effectively inhibit in vivo HIV replication [120], and that a mutated form of APOBEC3G that does not bind to Vif performs significantly better at suppressing HIV replication compared to other drugs [121]. Recently, Kurusu et al. used an extension of the standard model Equation (1) with humanized mouse data to investigate, for the first time, how APOBEC3G affects viral kinetics in vivo and showed that, indeed, APOBEC3G robustly restricted the initial stages of viral growth [122], confirming the therapeutic prospects of APOBEC3G.

Another study demonstrating the importance of incorporating viral intracellular detail into models of HIV infection, this time outside of direct therapeutic applications, was provided by Althaus et al. [123]. Briefly, an extension of the standard model Equation (1) to account for transcriptional subclasses of HIV-1-infected cells (checkpoint ➃ in Figure 2) was validated against data from five HIV-infected study participants following initiation of ART. The model showed that the pool of latently infected cells becomes rapidly established during the first months of acute infection and only slowly increases afterwards, an essential result for the investigation of an HIV cure.

As mentioned before, these examples are not meant as an exhaustive list of applications. There are many other notable examples of insights gained through explicit modeling of intracellular infection stages, with a few listed here [6,80,81,123,124,125,126,127,128,129].

### 5.2. Intracellular Modeling of HCV Infection and Therapy

In one of the first mathematical models of intracellular interactions in hepatitis C virus infection, Dahari et al. developed a nine-dimensional ODE system for virus–host dynamics of subgenomic HCV replication in vitro from transfection to steady state (checkpoints ➁–➄ in Figure 2). Qualitative investigation of the model showed that HCV RNA replication occurring in a membrane compartment has advantages for the HCV life cycle [130]. While these complex models explained previously unaddressed molecular interactions, there was little data available for their validation. An opportunity to address detailed interactions arose with the development of antiviral therapies. The role of IFN-α therapy was considered in the context of the large mathematical model in [130], in order to determine its potential mode of action, e.g., blocking HCV protein production (checkpoint ➄ in Figure 2), blocking RNA synthesis, or enhancing RNA degradation (checkpoint ➃ in Figure 2). Comparison of the model to HCV RNA titers showed that IFN-α therapy results in lower protein production and RNA synthesis [131].

**Figure 4:**
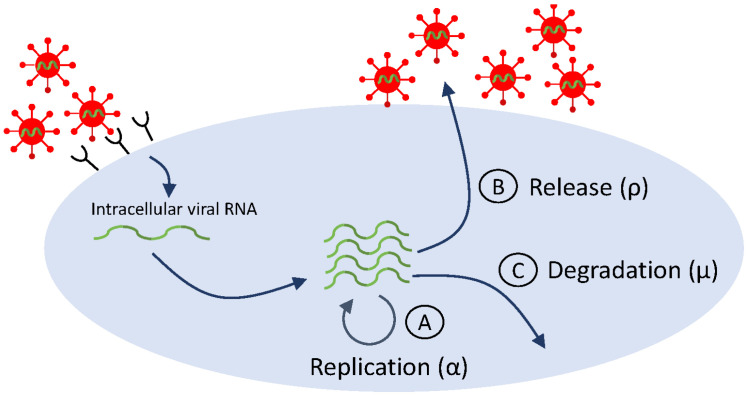
Simplified life cycle diagram used to model DAA therapy in HCV infection via Equation (6).

Similar to early HIV therapy, treatment of hepatitis C virus infection with interferon-α (INF-α) has shown consistent patterns in viral dynamics among patients: delayed viral response, followed by a biphasic decay in HCV RNA. Data fitting to HCV RNA titers from INF-α treatment trials resulted in viral clearance estimates of c=6.2±1.8 per day (corresponding to a serum half-life $t_{1/2}=2.7$ h) [18]. With the development of directly acting antivirals [132,133] (which revolutionized HCV therapy and led to HCV cure [134]), the accuracy of basic model estimates came into question when the fits of the basic model Equation (2) to HCV RNA data resulted in clearance rate estimates of c=23.3 per day (corresponding to half-life of 45 min), six times faster than the estimated viral clearance under IFN-α therapy [39]. This large difference is puzzling, since virus clearance rate should not be dependent on therapy. Guedj et al. hypothesized that the differences are due to intracellular dynamics, which may be triggered by DAA. To address the intracellular effects of DAA, model Equation (2) was extended to incorporate features of the viral life cycle (see Figure 4, which is a simplification of Figure 2). The extended model assumes interactions between target hepatocytes T(t) at time *t*, infected hepatocytes I(t,a) at time *t* and age of infection *a*, intracellular viral RNA (vRNA) R(t,a) at time *t* and age of infection *a*, and virus V(t) at time *t*. vRNA is produced at rate $\alpha e^{-\gamma t}$, degraded at rate μ, and exported from the cell at rate ρ (checkpoints Ⓐ, Ⓑ, and Ⓒ in Figure 4, respectively). DAA-therapy has three intracellular effects: blocking intracellular RNA production by a fraction $\left(1-\epsilon_{a}\right)$, blocking vRNA packaging/secretion by a fraction $\left(1-\epsilon_{s}\right)$, and enhancing the vRNA degradation rate μ by a factor κ≥1. All other interactions and parameters are as before. The new model is:

(6) $$\begin{array}{cc} \frac{dT}{dt} & =\lambda -dT-(1-\eta )\beta TV,\\ \frac{\partial I}{\partial t}+\frac{\partial I}{\partial a} & =-\delta I(t,a),\\ \frac{\partial R}{\partial t}+\frac{\partial R}{\partial a} & =\left(1-\epsilon_{a}\right)\alpha e^{-\gamma t}-\kappa \mu R\left(t,a\right)-\left(1-\epsilon_{s}\right)\rho R\left(t,a\right),\\ \frac{dV}{dt} & =\left(1-\epsilon_{s}\right)\rho \int_{0}^{\infty }R\left(t,a\right)I\left(t,a\right)da-cV,\\ I(0,t) & =\beta V\left(t\right)T\left(t\right),I\left(a,0\right)=I_{0}\left(a\right),R\left(0,t\right)=1,R\left(a,0\right)=R_{0}\left(a\right). \end{array}$$

Using the method of characteristics, Rong et al. [45] made assumptions that reduced the solution of Equation (6) to a sum of three exponential terms:

(7) $$V\left(t\right)=V_{0}\left(Ae^{-ct}+Be^{-\lambda t}+Ce^{-\delta t}\right),$$

where $\lambda =k\mu +\rho (1-\epsilon_{s})+\delta$. The solution of Equation (7) gave several biological explanations for the observed patterns of viral decline under different therapies. In particular, it showed that the biphasic decline during INF-α therapy is given by slopes λ and δ and the biphasic decline during DAA therapy is given by slopes *c* and λ. This reconciles estimates among studies, and corrects the estimate for HCV clearance rate to c=23.3 per day (corresponding to half-life $t_{1/2}=45$ min). This is an example in which adding complexity, in the form of intracellular viral RNA, was necessary in order to explain the data and obtain consistent parameter estimates.

### 5.3. Intracellular Modeling of HBV Infection and Therapy

In the case of hepatitis B virus infection, mathematical models described by ordinary differential equations have been extensively used to investigate various aspects of the intracellular infection process [135,136,137]. In particular, they explored the role of HBsAg production from integrated DNA in the process of HBV infection (checkpoint ➄ in Figure 3) [138,139], they helped distinguish between the kinetics of the noncytolytic and cytolytic immune responses during acute HBV infection [140], and they investigated the impact of e-antigen (HBeAg) and HBsAg on inducing immunological tolerance during HBV infection [136,141]. Recently, a modified version of model Equation (6) (developed to study the effects of drugs on HCV infection) was adapted in order to describe the recycling of the intracellular covalently closed circular DNA (cccDNA) (checkpoint ➇ in Figure 3). By applying such multiscale mathematical models, the amount and dynamics of intrahepatic cccDNA were quantified [142].

Importantly, mathematical models of viral life cycle can help identify antiviral targets, predict antiviral modes of action, and optimize interventions in hepatitis B viral infections. Early treatment involved monotherapy with IFN-α and combination therapy with pegylated IFN-α and nucleos(t)ide analogues (NUCs), the only therapies currently approved by FDA. Pegylated IFN-α activates the immune system, and, hence, cannot be administrated for a long period of time. The NUCs block reverse transcription (checkpoint ➆ in Figure 3) and are well tolerated. The HBV DNA under these therapies follows biphasic patterns of decay in most patients (as in HIV and HCV), and, hence, can be described using the standard model Equation (2). In a few patients, however, the HBV DNA follows a triphasic decay. To account for the additional dynamic, logistic growth terms were added to both uninfected and infected cell populations in model Equation (2), as follows:

(8) $$\begin{array}{cc} \frac{dT}{dt} & =\lambda -dT+r_{T}T\left(1-\frac{T+I}{K}\right)-\left(1-\eta \right)\beta TV,\\ \frac{dI}{dt} & =r_{T}T\left(1-\frac{T+I}{K}\right)+\left(1-\eta \right)\beta TV-\delta I,\\ \frac{dV}{dt} & =(1-\epsilon )pI-cV, \end{array}$$

where *K* is the liver carrying capacity and $r_{T}$ and $r_{I}$ are the uninfected and infected cells per capita growth rate. Data fitting has predicted that the entire liver needs to be infected at the start of therapy for model Equation (8) to have triphasic decay as one of its outcomes [24,143].

While pegylated IFN-α and NUCs suppress virus replication, they rarely lead to clearance of serum HBV DNA and HBsAg [144]. Compounds such as RNA interference and nucleic acid polymers, who are disrupting various stages of the HBV life cycle including entry, transcription, and assembly (checkpoints ➀, ➂, ➈ in Figure 3), are currently in (pre-)clinical trials. They show improved efficacy in reducing HBsAg (reviewed in [145]). To identify their efficacy and mechanism of action, expansion of model Equation (2) was created. They include additional variables for viral proteins such as HBsAg and HBeAg [138], for antibody against HBsAg [92], and for the pharmacokinetic of the drugs [138]. Fitting of these extended models to HBV DNA, HBsAg, HBeAg, and/or anti-HBs titers provided preliminary insights into the efficacy and MOA of these novel drugs and identified an early HBsAg kinetic response pattern that is associated with functional cure [146]. This is another example where adding complexity is essential to addressing therapeutic effects in decreasing viral proteins.

## 6. Conclusions

In-host models have been essential for understanding the dynamics of virus infection inside an infected individual. They have been used to gain knowledge on virus life cycle, intracellular and cellular virus–host interactions, and to provide inferences on the drug efficacy and mode of action.

The standard model of in-host virus infections is based on deterministic ODE models that only consider interactions between the target cells and the virus. In this review, we presented the usefulness of the standard model in determining viral characteristics. We then highlighted situations where there is need for model expansion through added complexity. This can take the form of extra compartments for intracellular processes in the ODE models, development of DDE models for delayed intracellular events, or development of PDE models that connect cellular and intracellular interactions. We noted situations where inclusion of intracellular events led to improvement in parameter estimates, unification of conclusions, and provided guidance for targeted therapeutics. We observed how development of multiscale intracellular–cellular model approaches that have improved the understanding of HCV infection and therapy (leading to cure) have inspired modeling progress in HBV infection, where the intracellular processes of the HBV life cycle are now included. While these advances highlight the importance of adding complexity (at the intracellular level) in our modeling endeavors, they are only powerful when they can be validated against empirical data. While we wait for advances in intracellular data collection, we should be mindful of which model we use in order to obtain an unbiased prediction given the available data. Performing sensitivity analyses [147,148,149], model identifiability [34,150,151], uncertainty quantification [152], and model reduction [153,154] allows modelers to determine the proper tradeoff between model complexity and data availability. Lastly, close interaction between modelers and experimentalists is needed in order to determine the type, amount, and frequency of data needed at each level of resolution in order to justify inclusion of complexity in in-host models of virus infections.

## Data Availability

No data was generated as part of this review.

## Abbreviations

The following abbreviations are used in this manuscript:

HIV	human immunodeficiency virus
HBV	hepatitis B virus
HCV	hepatitis C virus
HDV	hepatitis D virus
$R_{0}$	basic reproduction number
MOI	multiplicity of infection
HBsAg	hepatitis B s-antigen
HBeAg	hepatitis B e-antigen
cccDNA	closed covalent circular DNA
ART	Antiretroviral therapy
PI	Protease inhibitors
RT	Reverse transcriptase inhibitors
DAA	Direct acting antivirals
INF	Interferon
MOA	mode of action
ODE	ordinary differential equations
DDE	delay differential equations
PDE	partial differential equations
